# Binary Alkali-Metal Silicon Clathrates by Spark Plasma Sintering: Preparation and Characterization

**DOI:** 10.3390/ma9070593

**Published:** 2016-07-19

**Authors:** Igor Veremchuk, Matt Beekman, Iryna Antonyshyn, Walter Schnelle, Michael Baitinger, George S. Nolas, Yuri Grin

**Affiliations:** 1 Max-Planck-Institut für Chemische Physik fester Stoffe, 01187 Dresden, Germany; Igor.Veremchuk@cpfs.mpg.de (I.V.); Iryna.Antonyshyn@cpfs.mpg.de (I.A.); Walter.Schnelle@cpfs.mpg.de (W.S.); Michael.Baitinger@cpfs.mpg.de (M.B.); 2 Department of Physics, California Polytechnic State University, San Luis Obispo, CA 93407, USA; mbeekman@calpoly.edu; 3 Department of Physics, University of South Florida, Tampa, FL 33620, USA

**Keywords:** spark plasma synthesis, Zintl phase, intermetallic clathrate

## Abstract

The binary intermetallic clathrates K_8-*x*_Si_46_ (*x* = 0.4; 1.2), Rb_6.2_Si_46_, Rb_11.5_Si_136_ and Cs_7.8_Si_136_ were prepared from *M*_4_Si_4_ (*M* = K, Rb, Cs) precursors by spark-plasma route (SPS) and structurally characterized by Rietveld refinement of PXRD data. The clathrate-II phase Rb_11.5_Si_136_ was synthesized for the first time. Partial crystallographic site occupancy of the alkali metals, particularly for the smaller Si_20_ dodecahedra, was found in all compounds. SPS preparation of Na_24_Si_136_ with different SPS current polarities and tooling were performed in order to investigate the role of the electric field on clathrate formation. The electrical and thermal transport properties of K_7.6_Si_46_ and K_6.8_Si_46_ in the temperature range 4–700 K were investigated. Our findings demonstrate that SPS is a novel tool for the synthesis of intermetallic clathrate phases that are not easily accessible by conventional synthesis techniques.

## 1. Introduction

**Figure 1:**
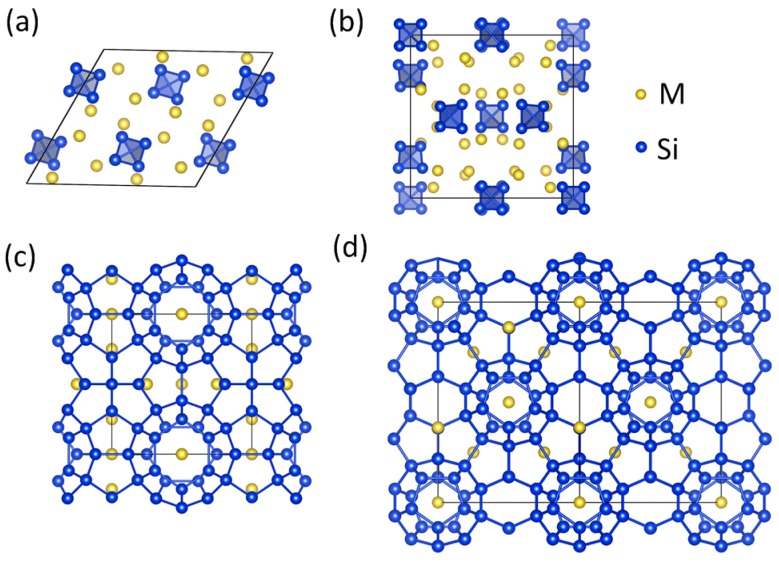
Crystal structures of (**a**) Na_4_Si_4_; (**b**) *M*_4_Si_4_ (*M* = K, Rb, or Cs); (**c**) *M*_8-*x*_Si_46_; and (**d**) *M*_24-*x*_Si_136_.

Binary phases of alkali metals with silicon have been prepared by thermal decomposition of the monosilicides *M*_4_Si_4_ (*M* = Na, K, Rb, Cs) for over six decades [1,2,3,4]. Structural investigations of the polycrystalline products revealed the existence of clathrate-I *M*_8-*x*_Si_46_ and clathrate-II *M*_24-*x*_Si_136_ silicides [5,6]. In both clathrate types, the alkali-metal atoms are enclosed in polyhedral cages formed in the tetrahedrally bonded silicon framework. In the clathrate-I crystal structure, the unit cell contains two Si_20_ and six Si_24_ cages, while in the clathrate-II structure there are sixteen Si_20_ and eight Si_28_ cages present in the unit cell. In the clathrate phases prepared by thermal decomposition the filling fraction of *M* in the silicon cages depends explicitly on both the reaction conditions and the size of the *M* atom relative to the size of the cage [6] (Figure 1).

Alternatively, redox techniques have been developed to obtain metastable clathrate phases [7,8,9,10] including Ge and Si allotropes with empty cages [11,12]. Defect-free single crystals of alkali metal silicon clathrates large enough for structure investigations have only been obtained in exceptional cases [4]. For example, crystals of K_8_Si_46_ were grown on *α*-Si exposed to potassium vapor [13], while crystals of ternary clathrate-II phases Rb_8_Na_16_Si_136_ and Cs_8_Na_16_Si_136_ form in closed ampoules directly from the melt [14,15]. Single-crystals suitable for measurement of intrinsic transport properties were first prepared by the electrochemically-driven transformation of Na_4_Si_4_ to Na_24_Si_136_ during the spark plasma treatment (SPS) [16,17]. Another approach—the kinetically controlled thermal decomposition (KCTD) method—can also provide relatively large single crystals [18,19]. Recently, a thermal decomposition at lower temperatures was employed for the preparation of Na_24-*x*_(Si*_y_*Ge_1-*y*_)_136_ and proved to be useful for the synthesis of silicon clathrates [20]. Extensions of the SPS and KCTD approaches have also recently reported, including use of multi-phase precursors or simultaneous ion-exchange and electrochemical reactions to prepare ternary or quaternary clathrates [21,22,23].

The unique advantage of SPS for the synthesis of clathrates by producing scalable, compact bulk materials also continues to be of great interest [4]. In this work we discuss both the preparation and consolidation of alkali-metal silicon clathrates by SPS. We report on the preparation and crystal structure of different clathrate phases (Table 1) as well as SPS densification of K_7.6_Si_46_ and K_6.8_Si_46_ powders by SPS that allow for the investigation of their transport properties over a large temperature range. The formation and evaporation the free alkali metals during the reaction allow us to work only with a small amount (less than 200 mg) of precursors and, in the results, after processing, we obtained 10–20 mg of the materials.

## 2. Results and Discussion

**Figure 2:**
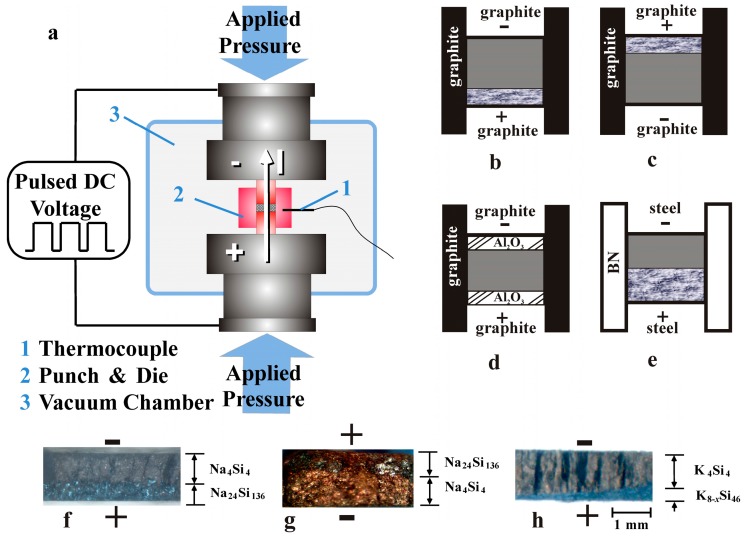
(**a**) SPS setup for the preparation of clathrates; (**b**–**e**) Location and relative amounts of the reaction products for different polarities and different die and punch materials; (**f**–**h**) Optical micrographs of the cross-sections from fractured post-reaction pellets illustrating the directional growth of clathrates at the anode.

As first described in reference [16], clathrates are formed during SPS redox-treatment by oxidation of Si_4_^4−^ at the anode while the alkali metal is reduced at the cathode (Figure 2a,b). Clathrate-II formation, according to

34 *M*_4_Si_4_ → *M*_24_Si_136_ + 112 *M*

can therefore be described by the half-reactions

34 Si_4_^4−^ − 112 e^−^ → Si_136_^24−^ (anode) 112 *M*^+^ + 112 e^−^ → 112 *M*^0^ (cathode) (1)

For clathrate-I formation according to

23 *M*_4_Si_4_ → 2 *M*_8_Si_46_ + 76 *M*

should be considered by means of the half-reactions

23 Si_4_^4−^ – 76 e^−^ → 2 Si_46_^8−^ (anode) 76 *M*^+^ + 76 e^−^ → 76 *M*^0^ (cathode) (2)

Reactions (1) and (2) describe the basics of the clathrate formation by SPS, whereby the process is driven by the DC field and the evaporation of the alkali metal at the cathode and its absorption by the graphite die (Figure 2b). In order to qualitatively investigate the influence of electric current on clathrate formation we performed three control experiments with Na_4_Si_4_ as the precursor. First, the polarity of the DC current was reversed, upon which it was found that the clathrate phase, Na_24_Si_136_ in this case, consistently forms at the anode (Figure 2c). In a second experiment, equal volumes of Al_2_O_3_ powder were placed above and below the precursor to create an electrically insulating barrier. The electrical current then presumably flowed through the low resistance graphite die such that the current passing through the Na_4_Si_4_ precursor is minimized. In this case no reaction was observed under the identical conditions used to form the clathrate Na_24_Si_136_ (Figure 2d). In the third experiment, the use of stainless steel punches combined with a die manufactured from boron nitride, a high electrical-resistivity material, presumably forced most of the current to pass through the precursor (as opposed to the surrounding die) (Figure 2e). A more rapid reaction than in the case of using the graphite die was observed. Optical photographs of cross-section of SPS-processed pellets (Figure 2f,h) clearly show that formation of the clathrate phases occurred at the anode during SPS processing. This was also the case for all clathrate compositions prepared in this study.

**Figure 3:**
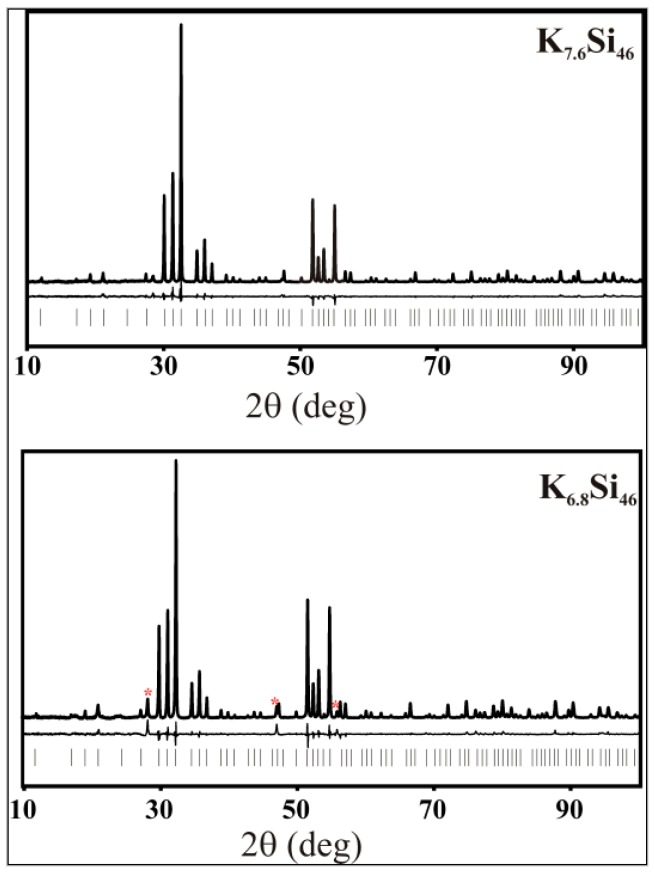
Powder X-ray diffraction (PXRD) patterns of the SPS-prepared clathrates-I K_7.6_Si_46_ (550 °C) and K_6.8_Si_46_ (600 °C). Red asterisks mark the reflections of *α*-Si (<0.2 mass % in K_7.6_Si_46_ and 1.5 mass % in K_6.8_Si_46_). The experimental intensities are shown as a solid line. The difference between the experimental and calculated intensities is shown below the experimental data; the tick marks represent the positions of the diffraction reflections of the clathrate-I phase.

**Figure 4:**
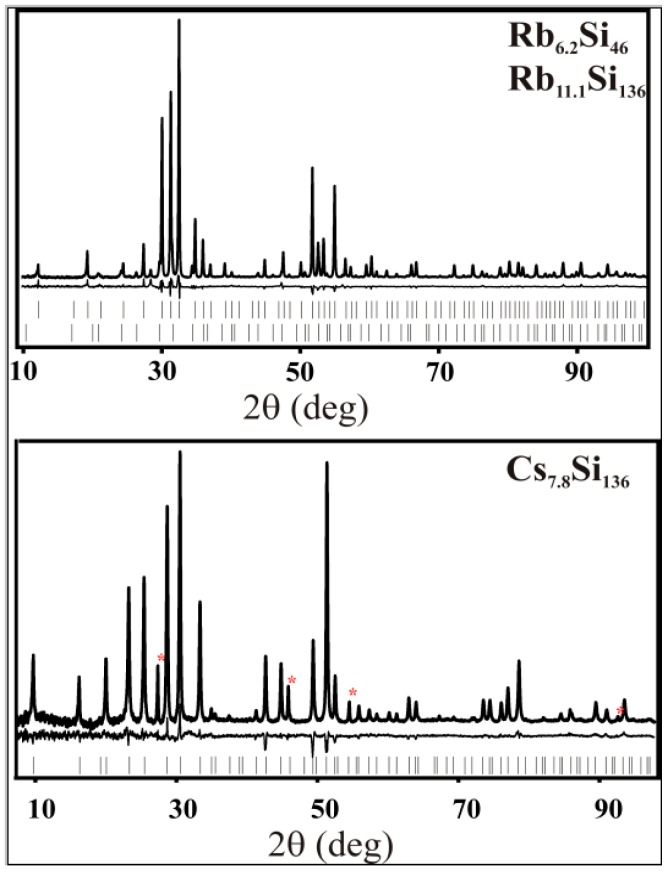
Powder X-ray diffraction (PXRD) patterns of the Rb_6.2_Si_46_ and Rb_11.1_Si_136_ mixed product and clathrate-II Cs_7.8_Si_136_. The experimental intensities are shown as a solid line. The differences between the experimental and calculated intensities are shown below the experimental data. The tick marks represent the positions of the diffraction reflections. Red asterisks mark the reflections from *α*-Si (<0.2 mass % in Rb_6.2_Si_46_ and Rb_11.1_Si_136_ and 2.5 mass % in Cs_7.8_Si_136_).

The crystal structures of the SPS-prepared clathrate phases were investigated by the Rietveld method using powder X-ray diffraction (PXRD) data. Experimental, calculated, and difference diffraction patterns for the clathrate-I and II samples are shown on Figure 3 and Figure 4, respectively, and the resulting crystallographic data are presented in Table 2 and Table 3, respectively.

The lattice parameters for K_7.6_Si_46_, Rb_6.2_Si_46_, and Cs_7.8_Si_136_ show good agreement with those synthesized by other preparation methods [7,16,18,19,24,25,26]. The lattice parameter for Rb_11.5_Si_136_, as expected, has an intermediate value between that reported for K_17.2_Si_136_ [19] and Cs_8_Si_136_ [6]. In all cases the silicon framework sites were found to be fully occupied (no vacancies), in agreement with prior results for binary silicon clathrates [7,16,18,19,26,27,28]. The stable covalent Si–Si bonds in silicon clathrates allow for a large excess of electrons that fill antibonding states. In a rigid-band model, a high concentration of conduction electrons are provided by charge transfer from the alkali-metal guests, thus inducing metallic behavior, as confirmed experimentally [15,29]. In contrast to SPS-prepared Na_8_Si_46_ and Na_24_Si_136_, which show full occupancy of the polyhedral cages by sodium [19], the SPS-prepared clathrates of potassium, rubidium and caesium exhibit partial occupancy of the alkali-atom positions (the specific refined compositions are given in Table 2 and Table 3).

**Figure 5:**
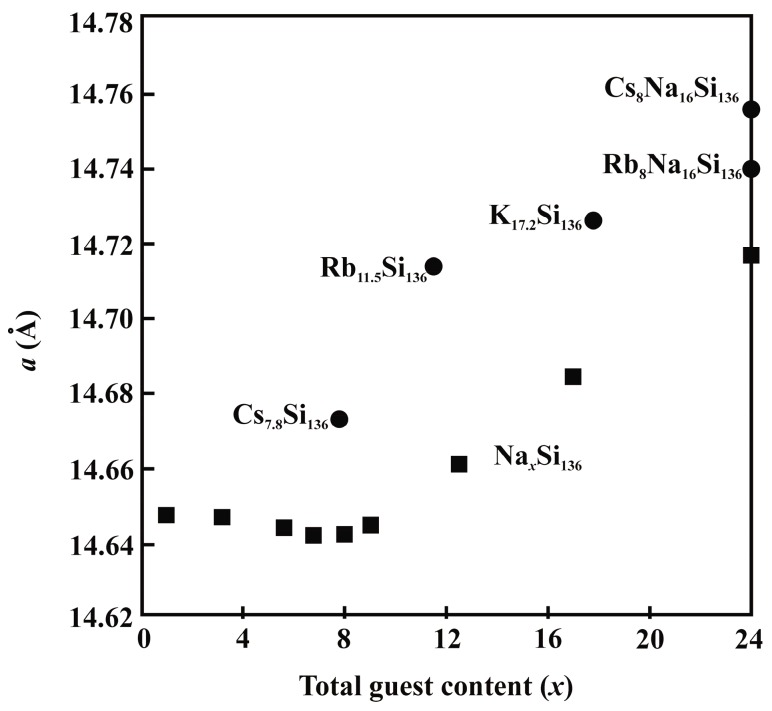
Lattice parameters versus *M* content, *x* for clathrate-I Na*_x_*Si_136_ (0 < *x* < 24) [28], Na_24_Si_136_ [16], K_17.2_Si_136_ [19], Rb_11.5_Si_136_ (this work), and Cs_7.8_Si_136_ (this work), as well as ternary Rb_8_Na_16_Si_136_ and Cs_8_Na_16_Si_136_ [27].

The compositions of clathrates-II prepared in this study together with previously reported in the literature [16,19,29,30] allow for a comparison of the lattice parameter versus guest-atom content in clathrate-II phases. As shown in Figure 5, in all cases the lattice parameter increases with those increasing *M* content in the Si_20_ (crystallographic site 16*c*) cage. In addition, the lattice parameter also increases with increasing alkali-ion radius [31] for a specific *M* concentration. Preferential occupancy of the larger Si_28_ (crystallographic site 8*b*) cages was observed for the case of Rb_11.5_Si_136_, as well as for for Na*_x_*Si_136_ [30,32].

**Figure 6:**
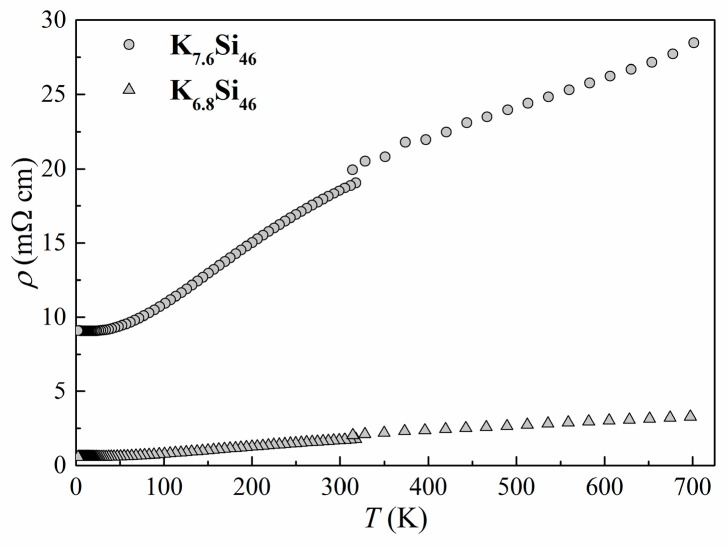

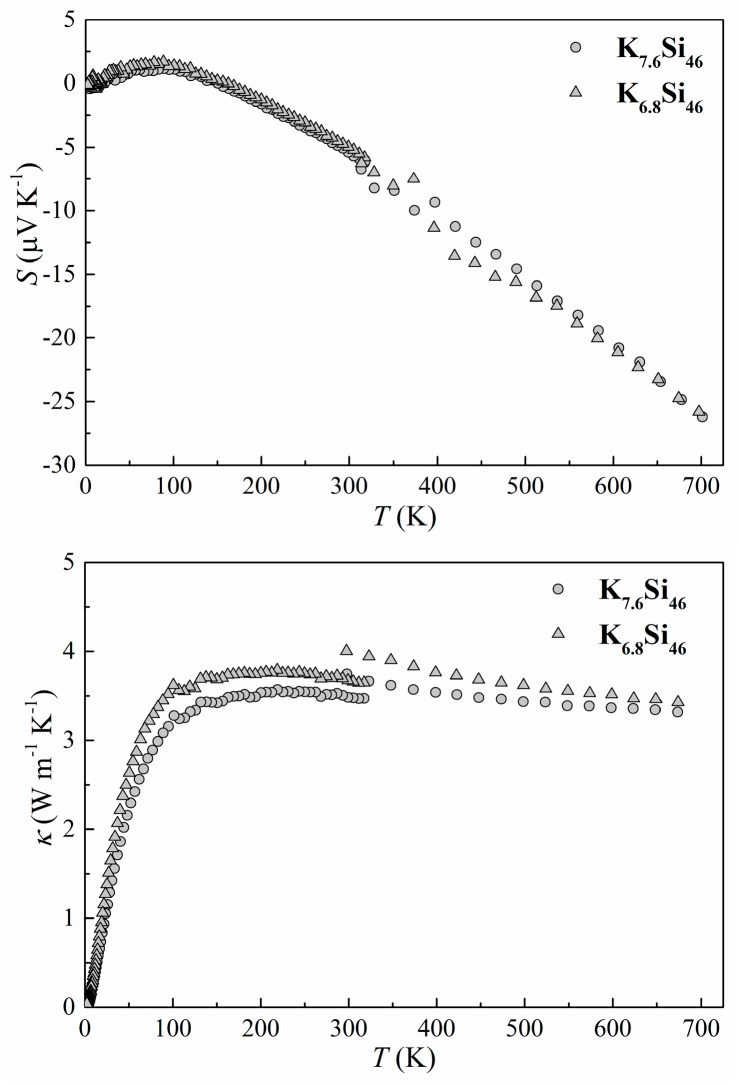
Transport properties of polycrystalline K_7.6_Si_46_ and K_6.8_Si_46_. The low temperature, 2 to 300 K, and high temperature, 300 to 700 K, are described in the text. (**Top**) electrical resistivity *ρ*; (**Middle**) Seebeck coefficient *S*; (**Bottom**) thermal conductivity *κ*.

In order to further characterize the products formed by SPS we measured the electrical and thermal transport properties of K_7.6_Si_46_ and K_6.8_Si_46_. The density of the pellets (only K_8-*x*_Si_46_) was approximately 90% of the calculated density based on PXRD data. Temperature-dependent electrical resistivity *ρ* (Figure 6, top), Seebeck coefficient *S* (Figure 6, middle), and thermal conductivity *κ* (Figure 6, bottom) in the temperature range from 10 to 700 K are shown in Figure 6. The agreement between the high- and low-temperature data, measured with two different setups on two different specimens, is an indication of the homogeneity of the materials prepared by SPS. Both materials K_7.6_Si_46_ and K_6.8_Si_46_ show metallic temperature dependence of *ρ* and *S*, in agreement with the seminal work by Cros et al. [6]. The fact that these materials show metallic conduction follows from the simple assertion that each K atom donates one electron to the silicon framework resulting in an overall high electron concentration. Measured *ρ* of cold-pressed K_7.6_Si_46_, obtained from the thermal decomposition of K_4_Si_4_, was reported to be much higher: *ρ* ~ 140 mΩ·cm at room temperature with weak temperature dependence [28]. The large discrepancy between those results and the data shown in Figure 6 may be attributed to the microstructure of the specimens and the typically poor inter-granular contact that is achieved using cold pressing [28]. Nevertheless, the *ρ* values shown in Figure 6 are significantly larger than expected for the presumed high electron concentration. In addition, the residual resistance ratio, *ρ*_300K_/*ρ*_10K_ = 3.6, is also relatively low for a good metal and significantly smaller than the values obtained for single crystals of Na_8_Si_46_ (*ρ*_300K_/*ρ*_10K_ = 36) [33] and Na_24_Si_136_ (*ρ*_300K_/*ρ*_10K_ = 14) [17]. These results reiterate the need for high-density polycrystalline specimens in order to minimize the effects of grain boundary scattering on the transport properties of intermetallic clathrates [34].

## 3. Materials and Methods

### 3.1. Synthesis of Precursors

Binary precursors *M*_4_Si_4_ (*M* = Na, K, Rb, or Cs) were prepared from alkali metal pieces (ChemPur, Karlsruhe, Germany 99.9 wt %) and coarsely ground silicon (ChemPur, 99.999 wt %), by sealing of the precursor mixture under argon in small, welded tantalum ampoules that were enclosed under vacuum inside silica tubes and heated to 800 °C at an average rate of 3 °C/min. The ampoules were held at 800 °C for 4 h before being slowly cooled to room temperature (furnace cooled). The reaction products were determined to be single phase *M*_4_Si_4_ by powder X-ray diffraction (PXRD). All sample manipulations were performed in an argon-filled glove box (H_2_O < 1 ppm; O_2_ < 1 ppm). **CAUTION**: *M*_4_Si_4_ compounds are sensitive to air and moisture (pyrophoric) and must be kept under inert atmosphere at all times.

### 3.2. Spark Plasma Synthesis

SPS experiments were conducted under vacuum (background pressure <10 Pa) using a dedicated SPS setup in a protective argon atmosphere (Figure 2a; SPS: 515 ET, Syntex-Fuji, Japan; glove box: MBraun, Garching, Germany) [24]. Table 1 contains the preparation conditions for the clathrates synthesized within this study. The precursors were ground to fine powder and loaded into graphite or boron nitride (*h*-BN) dies with an inner diameter of 8 mm. Graphite or stainless steel punches were used to apply the uniaxial pressure. Tantalum foil was employed to surround the powder specimen on all sides, isolating the specimen from the die and punches during SPS processing. Graphite dies worked well for Na, however they were observed to react with alkali-metal vapor for precursors of K, Rb, and Cs and often cracked the graphite die during SPS processing. For these alkali metals *h*-BN was found to be most suitable. After SPS the products were isolated from the residual precursor by washing first with ethanol and then distilled water under flowing argon.

### 3.3. Sample Characterization

Powder X-ray diffraction (PXRD) data were collected using the Guinier technique (Huber G670 camera, Rimsting, Germany, Cu-*K_α_*_1_ radiation, *λ* = 1.540598 Å, graphite monochromator, 5° ≤ 2*θ* ≤ 100°, ∆2*θ* = 0.005°). Crystal structure refinements were performed using the software WinCSD [25]. For the transport measurements, the K_7.6_Si_46_ and K_6.8_Si_46_ powders were first washed then consolidated by SPS using tungsten carbide tool (8 mm inner diameter) at 300 °C and a uniaxial pressure of 600 MPa under vacuum (background pressure of approximately 10 Pa) for 10 min. The density of the pellets was approximately 90% of the calculated density based on PXRD data. The specimens were cut with a diamond wire saw into parallelepiped pieces 1.5 mm × 1.5 mm × 8.0 mm for electrical transport measurements and low-temperature thermal conductivity measurements, and 1.5 mm thick and 8 mm in diameter disks for thermal diffusivity measurements. Electrical resistivity (*ρ*) and Seebeck coefficient (*S*) measurements from 22 to 450 °C on SPS densified K_7.6_Si_46_ and K_6.8_Si_46_ specimens were performed with a commercial ZEM-3 setup (ULVAC-RIKO, Munich, Germany). Specific heat (C*_p_*) measurements were performed on a Netzsch Pegasus DSC (Selb, Germany) while heating to 450 °C at a heating rate of 10 °C/min in an Ar atmosphere. Thermal diffusivity, *D*, was measured from 22 °C to 450 °C using the laser-flash technique (LFA 457 Micro Flash, Netzsch, Selb, Germany). The temperature dependent thermal conductivity, *κ*, was calculated using the relation *κ* = *C_p_* × *D* × *d* where *d* is the density in g/cm^3^. Low temperature *ρ*, *S*, and *κ* data were collected using the TTO-option (Thermal Transport Option) of a PPMS (Physical Property Measurement System) (Quantum Design, San Diego, CA, USA) using a four-point method.

## 4. Conclusions

Binary intermetallic clathrates K_8-*x*_Si_46_ (*x* = 0.4; 1.2), Rb_6.2_Si_46_, Rb_11.5_Si_46_, and Cs_7.8_Si_136_ were synthesized by spark plasma synthesis from the respective *M*_4_Si_4_ precursors. The formation of polycrystalline products at the anode of the SPS setup was observed for all compounds. The preparation of the new clathrate-II phase Rb_11.5_Si_136_ is further evidence that SPS is useful for the inorganic synthesis. The role the electric field is in driving the chemical conversion of the precursor into the clathrate phase at the anode during SPS processing is emphasized. Conventional thermal decomposition (CTC) [6,28,35], kinetically controlled thermal decomposition (KCTD) [18,19], chemical oxidation (CO) [7,27], and SPS yield different intermetallic clathrate phases from the same *M*_4_Si_4_ precursor (Table 4). While the influence of temperature clearly plays a role in clathrate formation, a definitive understanding of the local formation mechanisms in each of these synthetic approaches [36] is of great interest and is an ongoing task.

## Figures and Tables

**Table 1:** Spark-plasma synthesis (SPS) conditions for the silicon clathrates.

Precursor	*T_R_* (°C) *^a^*	*t_R_* (min) *^b^*	Die/Punches *^c^*	Products *^d^*
Na_4_Si_4_	550	180	C/C	Na_24_Si_136_ (M) + Na_8_Si_46_ (m)
	600	180	C/C	Na_24_Si_136_ (M)
	700	180	C/C	Na_24_Si_136_ (M) + Si (m)
K_4_Si_4_	500	60	BN/SS	K_8-*x*_Si_46_ (tr)
	550	60	BN/SS	K_8-*x*_Si_46_ (M)
	600	60	BN/SS	K_8-*x*_Si_46_ (M) + Si (m)
	650	60	BN/SS	Si (M) + K_8-*x*_Si_46_ (m)
Rb_4_Si_4_	450	60	BN/SS	Rb_8-*x*_Si_46_ (M) + Rb_24-*x*_Si_136_ (m) + Si (tr)
	500	60	BN/SS	Si (M) + Rb_8-*x*_Si_46_ (m) + Rb_24-*x*_Si_136_ (m)
Cs_4_Si_4_	350	60	BN/SS	Cs_8-*x*_Si_136_ (M) + Si (tr)

*^a^* *T_R_* is the reaction temperature; *^b^* *t_R_* is the treatment time; *^c^* Materials for the die and punches: C = graphite, BN = *hp*-boron nitride, SS = stainless steel; *^d^* Excluding unreacted precursor. M = majority phase, m = minority phase, tr = trace.

**Table 2:** Crystallographic information on the clathrate-I phases prepared by SPS (PXRD data).

Phase	K_7.6(1)_Si_46_	K_6.8(1)_Si_46_	Rb_6.2(1)_Si_46_
Lattice parameter *a*, Å	10.2776 (1)	10.2747 (1)	10.2848 (1)
Radiation, wavelength *λ*, Å	Cu *K_α_*_1_, 1.54056		
Maximal diffraction angle 2*θ*, °	100.30		
Residuals *R_I_*/*R_P_*	0.04/0.10	0.04/0.09	0.02/0.10
*M*1 2*a* (0 0 0)	Occ * = 0.783 (1)	Occ = 0.695 (4)	Occ = 0.244 (3)
*M*2 6*d* (¼ ½ 0)	Occ = 1.013 (3) **	Occ = 0.918 (3)	Occ = 0.961 (2)
Si1 6*c* (¼ 0 ½)	–	–	–
Si2 16*i* (*x x x*)	*x* = 0.1845 (1)	*x* = 0.1846 (1)	*x* = 0.1840 (1)
Si3 24*k* (0 *y z*)	*y* = 0.3068 (1)	*y* = 0.3060 (1)	*y* = 0.3043 (1)
	*z* = 0.1183 (1)	*z* = 0.1185 (1)	*z* = 0.1190 (1)

*: Occ—occupancy factor; **: The value 1.013 (3) for the K2 occupancy in K_7.6_Si_46_ reflects the real error of the refinement (≌4 e.s.d.) and helps to understand the reliability of the occupancy values for other clathrates.

**Table 3:** Crystallographic information on the clathrate-II phases prepared by SPS (PXRD data).

Phase	Rb_11.1(1)_Si_136_	Cs_7.8(1)_Si_136_
Lattice parameter *a*, Å	14.7142 (9)	14.6733 (3)
Radiation, wavelength *λ*, Å	Cu *K_α_*_1_, 1.54056	
Maximal diffraction angle 2*θ*, °	100.30	
Residuals *R_I_*/*R_P_*	0.10/0.10	0.05/0.13
*M*1 8*b* (⅜ ⅜ ⅜)	Occ * = 0.85 (2)	Occ = 0.968 (2)
*M*2 16*c* (0 0 0)	Occ = 0.272 (1)	Occ = 0.0
Si1 8*a* (⅛ ⅛ ⅛)	–	–
Si2 32*e* (*x x x*)	*x* = 0.2198 (6)	*x* = 0.2168 (2)
Si3 96*g* (*x x z*)	*x* = 0.1839 (2)	*x* = 0.1822 (1)
	*z* = 0.3698 (6)	*z* = 0.3699 (2)

*: Occ—occupancy factor.

**Table 4:** Clathrate phases obtained by different synthesis techniques from *M*_4_Si_4_ precursors.

Precursor	Preparation Technique *^a^*	Clathrate Phases Obtained	References
Na_4_Si_4_	CTC	Na_8_Si_46_ and Na_24-*x*_Si_136_	[6,33]
	KCTD	Na_8_Si_46_ and Na_24-*x*_Si_136_	[18]
	CO	Na_8_Si_46_ and Na_24-*x*_Si_136_	[7,25]
	SPS	Na_8_Si_46_ and Na_24-*x*_Si_136_	[16,24], this work
K_4_Si_4_	CTC	K_8-*x*_Si_46_	[6,26]
	KCTD	K_8-*x*_Si_46_ and K_24-*x*_Si_136_	[19]
	CO	K_8-*x*_Si_46_	[7]
	SPS	K_8-*x*_Si_46_	this work
Rb_4_Si_4_	CTC	Rb_8-*x*_Si_46_	[6,26]
	SPS	Rb_8-*x*_Si_46_ and Rb_24-*x*_Si_136_	this work
Cs_4_Si_4_	CTC	Cs_24-*x*_Si_136_	[6]
	SPS	Cs_24-*x*_Si_136_	this work

*^a^* CO = chemical oxidation, KCTD = kinetically controlled thermal decomposition, CTC = conventional thermal decomposition, SPS = spark plasma synthesis.
